# A Proteomic View of Cellular and Molecular Effects of Cannabis

**DOI:** 10.3390/biom11101411

**Published:** 2021-09-27

**Authors:** Morteza Abyadeh, Vivek Gupta, Joao A. Paulo, Veer Gupta, Nitin Chitranshi, Angela Godinez, Danit Saks, Mafruha Hasan, Ardeshir Amirkhani, Matthew McKay, Ghasem H. Salekdeh, Paul A. Haynes, Stuart L. Graham, Mehdi Mirzaei

**Affiliations:** 1 ProGene Technologies Pty Ltd., Macquarie Park, Sydney, NSW 2113, Australia; mabyadeh94@gmail.com; 2 Macquarie Medical School, Faculty of Medicine, Health and Human Sciences, Macquarie University, North Ryde, Sydney, NSW 2109, Australia; nitin.chitranshi@mq.edu.au (N.C.); angela.godinez@hdr.mq.edu.au (A.G.); danit.saks@hdr.mq.edu.au (D.S.); stuart.graham@mq.edu.au (S.L.G.); 3 Department of Cell Biology, Harvard Medical School, Boston, MA 02115, USA; Joao_Paulo@hms.harvard.edu; 4 School of Medicine, Deakin University, Geelong, VIC 2600, Australia; veer.gupta@deakin.edu.au; 5 School of Life and Environmental Sciences, University of Sydney, Sydney, NSW 2006, Australia; mafruha.hasan@sydney.edu.au; 6 Australian Proteome Analysis Facility, Macquarie University, Sydney, NSW 2109, Australia; ardeshir.amirkhani@mq.edu.au; 7 Bowel Cancer and Biomarker Laboratory, Kolling Institute, Northern Clinical School, The University of Sydney, Sydney, NSW 2006, Australia; matthew.mckay@sydney.edu.au; 8 Department of Molecular Sciences, Macquarie University, Macquarie Park, Sydney, NSW 2109, Australia; hsalekdeh@yahoo.com (G.H.S.); paul.haynes@mq.edu.au (P.A.H.)

**Keywords:** cannabis, cannabinoids, marijuana, tetrahydrocannabinol, cannabidiol, proteomics

## Abstract

Cannabis (*Cannabis sativa*), popularly known as marijuana, is the most commonly used psychoactive substance and is considered illicit in most countries worldwide. However, a growing body of research has provided evidence of the therapeutic properties of chemical components of cannabis known as cannabinoids against several diseases including Alzheimer’s disease (AD), multiple sclerosis (MS), Parkinson’s disease, schizophrenia and glaucoma; these have prompted changes in medicinal cannabis legislation. The relaxation of legal restrictions and increased socio-cultural acceptance has led to its increase in both medicinal and recreational usage. Several biochemically active components of cannabis have a range of effects on the biological system. There is an urgent need for more research to better understand the molecular and biochemical effects of cannabis at a cellular level, to understand fully its implications as a pharmaceutical drug. Proteomics technology is an efficient tool to rigorously elucidate the mechanistic effects of cannabis on the human body in a cell and tissue-specific manner, drawing conclusions associated with its toxicity as well as therapeutic benefits, safety and efficacy profiles. This review provides a comprehensive overview of both in vitro and in vivo proteomic studies involving the cellular and molecular effects of cannabis and cannabis-derived compounds.

## 1. Introduction

**Figure 1:**
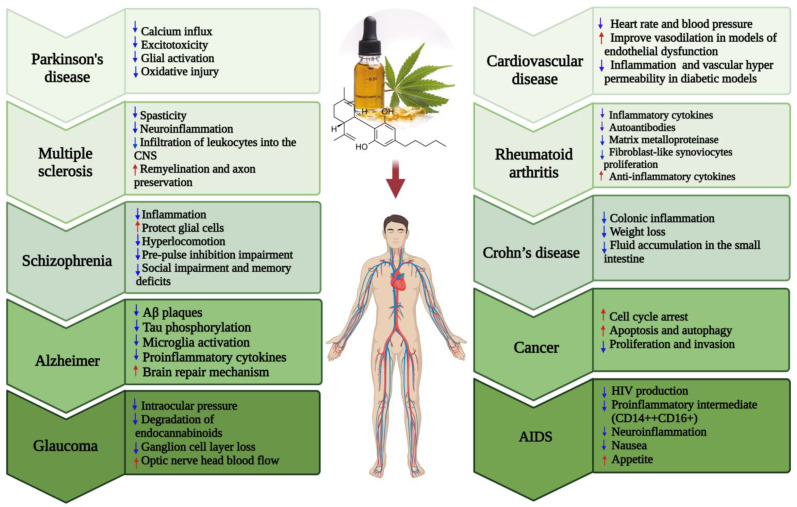
Various diseases against which cannabis/cannabinoids have potential for prevention and treatment (up and down arrows indicate increase and decrease respectively).

Cannabis is a generic term for *Cannabis sativa or Cannabis indica* plants or a group of bioactive compounds derived from these plants [1]. The plant parts and preparations have been used for thousands of years as a herbal medicine and for recreational purposes [2,3]. In the late 19th and early 20th centuries, it was known as “Devil’s lettuce” for its psychoactive effects and thus prohibited in many countries [4,5]. However, recent evidence has increasingly highlighted therapeutic potentials of cannabis chemical ingredients, termed cannabinoids, in a multitude of diseases such as Alzheimer’s disease (AD) [6,7,8], Parkinson’s disease (PD) [9,10], schizophrenia (SCZ) [11,12], multiple sclerosis (MS) [13,14], cancer [15], cardiovascular disease (CVD) [2], rheumatoid arthritis (RA) [16,17,18], Acquired Immunodeficiency Syndrome (AIDS) [19,20,21], glaucoma [22,23,24] and Crohn’s disease [25,26] (Figure 1). This has attracted considerable attention globally and multiple clinical trials have been designed to evaluate the therapeutic properties of various cannabinoids [27,28,29,30].

**Figure 2:**
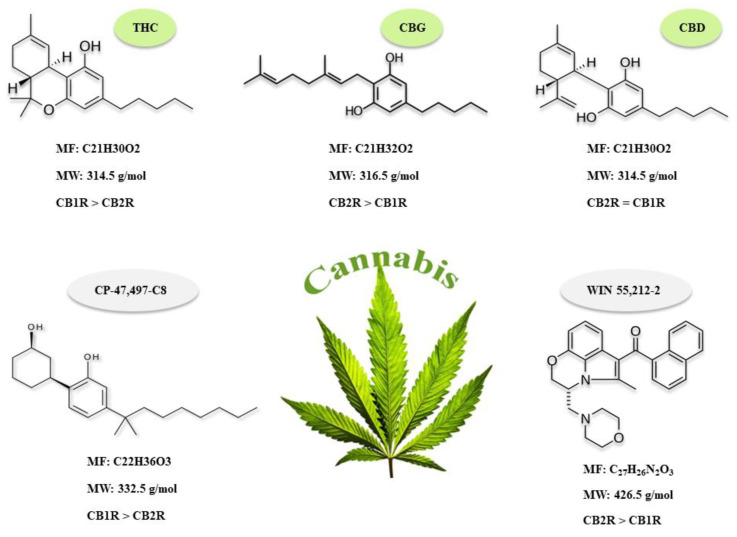
Chemical structures, molecular formula (MF), molecular weight (MW) and the affinity for CB1R and CB2R of the three natural components of cannabis including: Tetrahydrocannabinol (THC), Cannabidiol (CBD), Cannabigerol (CBG) and two synthetic cannabinoids known as CP-47,497-C8 and WIN 55,212-2.

Cannabis plants have been shown to contain 483 compounds with approximately 100 cannabinoids [31]. Of these, delta-9-tetrahydrocannabinol (THC) and cannabidiol (CBD) are the two most prominent cannabinoids which have received special attention for their therapeutic purposes (Figure 2) [32,33]. Currently, one cannabis-derived and three cannabis-related drugs are approved by the US Food and Drug Administration (FDA) including Epidiolex (cannabidiol) containing CBD, to treat tuberous sclerosis complex associated seizures, as well as Marinol (dronabinol), Syndros (dronabinol), and Cesamet (nabilone), all containing THC, which are used in the management of nausea and vomiting caused by chemotherapy and to increase appetite and prevent weight loss in patients with AIDS [34,35,36]. Another cannabis-based medication known as Sativex (nabiximols) that contains both THC and CBD, was developed by GW Pharmaceuticals and is currently approved in the UK and many European countries with applications to relieve the symptoms of MS such as neuropathic pain, spasticity, and overactive bladder [36,37]. Sativex has also received approval in Canada for the treatment of MS and cancer-associated pain. GW Pharmaceuticals recently granted Novartis exclusive rights to market Sativex in Australia, New Zealand and many countries in Asia, Middle East and the African sub-continent [37,38,39]. However, Sativex is not yet approved by the US FDA, with the agency indicating that there may be associated health risks, as there is limited research regarding its safety. Confusion, ideomotor slowing, fatigue, nausea and dizziness in MS patients, and nausea, vomiting, somnolence and dizziness in cancer patients are reported as some of the most prevalent side effects of Sativex [40,41].

The discovery of biological targets that are modulated by cannabis-derived components such as G protein-coupled receptors (GPCRs), including cannabinoid receptor 1 (CB1R), cannabinoid receptor 2 (CB2R), ion channels, and various nuclear receptors, has increased our understanding of how cannabis interacts with the human body [42,43,44]. However, the molecular mechanisms underlying the therapeutic properties of these components, and their after-effects, are poorly understood [45,46]. Cannabinoids are also synthesized endogenously within the body and act as agonists of Cannabinoid Receptors (CBRs), with examples of such endocannabinoids including N-arachidonoylethanolamine (AEA; anandamide) and 2-arachidonoylglycerol (2-AG) [47,48,49]. Collectively, endocannabinoids and their receptors, including lipid intermediates and enzymes that are involved in endocannabinoid synthesis and degradation, comprise the endocannabinoid system (ECS). The ECS plays a crucial role in the function of the brain, as well as takes a part in the regulation of endocrine and immune systems [47,48,50]. The ECS was identified to be impaired in various pathologies, but particularly in neurodegenerative conditions such as glaucoma, AD, PD and MS [50,51]. As such, its restoration via the administration of cannabis compounds has emerged as a promising therapeutic approach [52,53,54]. For instance, in glaucoma reduced levels of several endocannabinoids such as 2-AG and palmitoylethanolamide (PEA) in the ciliary body have been reported to have a profound effect in reducing intraocular pressure (IOP) [55,56,57,58]. Interestingly both systemic and topical administration of cannabinoids such as THC was shown to be effective in reducing the IOP, possibly through the same cannabinoid receptors present in the ciliary body and trabecular meshwork cells [59]. However, the duration of this IOP lowering effect is short and the exact underlying molecular mechanisms are still not well defined. In contrast, CBD treatment was shown to lead to a slight increase in IOP when administered at higher doses and thus may have a detrimental impact on glaucoma patients with high IOP [59]. A balance between levels of enzymes involved in the synthesis and degradation of endocannabinoids was shown to exhibit neuroprotective effects in traumatic, ischemic, inflammatory, and neurotoxic damage to the brain and may also play a similar protective effect on the retinal tissue [22,23,24]. Moreover, activation of CB1R and CB2R by synthetic or natural cannabinoid compounds was shown to be beneficial for memory improvement in the AD mouse model possibly through reducing the Aβ plaques, tau phosphorylation, microglia activation and proinflammatory cytokines, and also increasing the brain’s intrinsic repair mechanisms [6,7,8]. In addition, the protective effects of cannabinoid agonists in PD are mediated through reducing calcium influx, excitotoxicity, glial activation, and oxidative injury [9,10]. In MS, administration of endocannabinoids and their agonists decrease spasticity, neuroinflammation through inducing apoptosis and reducing the proliferation of T cells, promoting a reparative activation state of microglia and macrophages, decreasing pro-inflammatory cytokines levels, increasing anti-inflammatory cytokine production and inhibiting the infiltration of leukocytes into the CNS. Moreover, remyelination and axon preservation was also observed to occur upon exogenous administration of cannabinoids [13,14].

Gradual recognition of potential beneficial therapeutic effects of cannabis-derived compounds has allowed many countries to legalize its controlled use, which has generated renewed interest in cannabis research. Investigation into the relative adverse and beneficial effects of cannabis and its constituent cannabinoids at molecular levels is a research priority. Advances in high throughput technologies such as genomics, transcriptomics, proteomics and computational modeling have paved the way for better insights into the molecular pathways in disease and health. Proteomics studies, in particular, have attracted significant attention as they are able to determine protein abundance and regulation, changes, protein–protein interactions and impact on post-translational modifications [60,61,62,63]. Here, we provide a comprehensive literature review of proteomic studies performed in the field, with respect to the therapeutic and toxicological properties of cannabis.

## 2. Proteomic Studies on Cannabis

The therapeutic and toxic effects of cannabis have been extensively studied, however, most of the current research is focused on determining the toxicity profiles of cannabis-derived or related compounds [64]. The central nervous system (CNS) is the primary target of cannabis action, therefore most of the studies have investigated the effect of cannabis on the brain (Table 1). Cannabinoid receptors; mainly CB1R and to some extent CB2R are expressed throughout the hippocampus, amygdala, and cerebral cortex. Therefore, these brain regions are the primary targets for cannabis compounds and lead to instant psychoactive consequences such as memory and learning impairment; distorted perception, cognition and problem-solving ability; and anxiety symptoms. However unlike THC or cannabis extracts the second most abundant cannabis compound cannabidiol (CBD), does not exhibit psychotropic properties [65]. The association of cannabis with several mental disorders, particularly schizophrenia onset and progression, has been reported [66]. Interestingly, this association was also observed at the molecular level through proteomic analysis. A label-free proteomics study by Delgado-Sequera and colleagues (2020) compared the proteome of cells obtained from the olfactory neuroepithelium (ON) within the nasal cavity of cannabis users and healthy controls. Here, the ON cells were used as they can be easily and safely obtained from nasal brushing and reflect disease-related changes in neurons [67,68]. The study identified 65 differentially expressed proteins (DEPs), that were mainly related to the cytoskeleton (particularly microtubule dynamics and its influence on cell morphology), as well as cell proliferation, neurite outgrowth and apoptosis. Some of these changes were similar to the previously reported protein expression changes in schizophrenia (SCZ), bipolar disorder (BP) and DiGeorge syndrome (DGS) [69].

In another study, Barrera-Conde and colleagues (2021) determined the proteome changes of ON cells obtained from SCZ patients. The study was designed to include SCZ patients using cannabis (SCZ/c), SCZ patient non-consumers (SCZ/nc), healthy controls using cannabis (C/c) and healthy non-consumers (C/nc). Their investigation yielded 1185, 1209 and 1584 DEPs in C/c, SCZ/nc and SCZ/c respectively, compared to C/nc. There were 845 DEPs that were common between C/c, SCZ/nc and SCZ/c groups. The DEPs identified have mainly been implicated in regulating protein metabolism and immune system function. Amongst these, 102 DEPs were found exclusively in C/c compared to C/nc, and these proteins were predominantly associated with immune system function and RNA metabolism. Overall, their studies have established that cannabis affects the immune system and protein metabolism as the most common deregulated pathways between SCZ patients and cannabis users, as well as pathways pertaining to RNA metabolism, protein localization, and cellular signaling responses to external stimuli [70]. Taken together, these studies highlight the modulatory effects of cannabis on cellular function in the CNS, as well as its impact on the immune system and metabolism.

Apart from the CNS, both CB1R and CB2R are expressed in the human detrusor and urothelium, another major site of action of cannabis and associated compounds. The beneficial effects of cannabis on the urinary symptoms of MS patients have been previously reported in several epidemiological studies and clinical trials [71,72]. However, knowledge about the underlying molecular mechanisms is limited. Nedumaran and colleagues investigated the urinary proteome changes of cannabis users compared to healthy controls using liquid chromatography tandem mass spectrometry (LC-MS/MS). Their results identified 19 DEPs, including 14 up-regulated proteins that were principally related to lipid metabolism, immune response and inflammatory activity, and 5 down-regulated proteins implicated in intestinal and renal absorption, RNA and iron metabolism and neural differentiation. Interestingly, 91 proteins that are mainly involved in immunological and metabolic pathways were only found in the urine samples of cannabis users, while 46 proteins that related to the integrin signaling, inflammation, PI3 kinase pathway and nicotinic receptor signaling were depleted in the urine of cannabis users compared to the healthy controls [73]. Collectively, these results along with other studies may suggest an enhanced immune response in cannabis users against potential pathogens, leading to reduced incidence of urinary tract infections. The effects of cannabis on the immune system have been investigated in another proteomics study by Hinckley and colleagues, which explored the plasma proteome changes in eight discordant and four concordant twin pairs who either used or did not use cannabis within the last 30 days. These results provided evidence of differential regulation of 13 proteins, which were associated with regulating THC-COOH levels and immune system pathways, including cytokine-mediated signaling pathways involving CD86, CX3CL1, IL19, IL1RAPL2, IL23R and MYC proteins. Notably, most of these proteins and networks have previously been shown to be involved in T lymphocyte-mediated immunogenicity [74].

## 3. Proteomic Studies on Tetrahydrocannabinol

**Figure 3:**
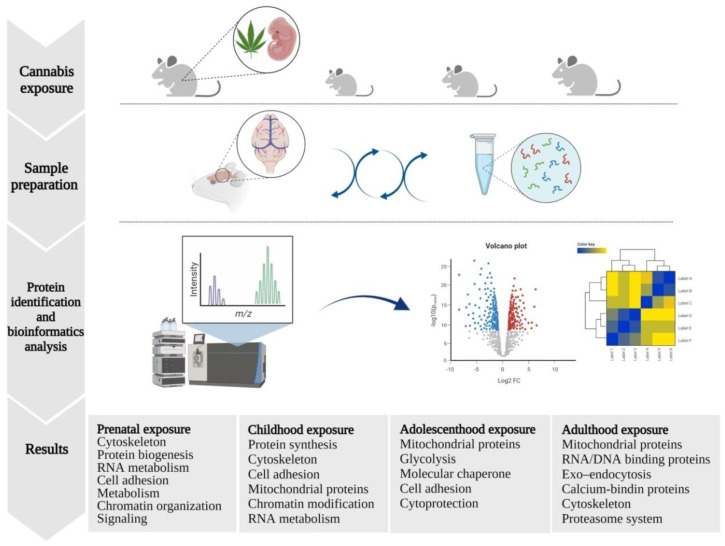
Proteomics workflow to identify differentially expressed proteins (DEPs), their function and associated pathways in the brain of mice/rats, following exposure to THC at different life stages.

THC is one of the major psychoactive ingredients of cannabis and is the focus of most research studies [86]. Although there are investigations into the therapeutic properties of THC, significant research has focused on the toxic effects of this compound, including proteomics studies (Figure 3). Bindukumar and colleagues (2008), using two-dimensional differential in-gel electrophoresis (2D-DIGE), showed the neuroprotective effects of THC exposure for 48 h on normal human astrocytes, mediated through the significant up-regulation of 24 proteins. The identified proteins were mainly involved in glycolysis, protein binding and folding, and kinase activity, as well as molecular chaperone activities of aldolase A (ALDOA), 70 kDa heat shock protein 5 (HSP70-5) and creatine kinase (CK). This study also suggested the potential therapeutic effects of cannabis on side-effects caused by HIV infection, with the up-regulation of glutathione peroxidase (GPX), which is ordinarily down-regulated in disease and promoting oxidative stress [75,87]. In a recent study, Xiao and colleagues (2021) have shown inhibitory effects of THC against Aβ induced cell cycle arrest and apoptosis in microglial cell cultures. Moreover in vivo THC administration reduced the Aβ burden in the hippocampus of 8 months old transgenic mouse model of AD and resulted in improved learning and memory. Furthermore proteomic analysis yielded 157 DEPs in hippocampus of THC treated AD mice model compared to the controls; of those 94 proteins were up-regulated that are predominantly involved in IFNγ production, T cell activation and lymphocyte activation. The, remaining 63 proteins were down-regulated and are related to protein oligomerization, protein binding and assembly and organization of membrane raft structures. Further analysis revealed the key roles of Ras/ERK signaling in THC therapeutic effects against microglia cell cycle arrest and apoptosis and suggested THC as a beneficial agent for alleviating AD progression [85].

In contrast to the above described potentially beneficial effects, prenatal exposure to cannabis was shown to adversely affect neonatal growth and brain development, leading to cognitive and behavioral impairments and attention deficit disorders [88,89]. The mechanisms underlying these toxic effects are ill-defined, although a proposed mechanism by Tortoriello and colleagues (2014) suggests that these effects may be mediated through disruption of endocannabinoid signaling and shrinkage of axonal growth and guidance [81]. In this study, pregnant mice received intraperitoneal injections of THC, followed by an analysis of brain tissue of the fetuses. The fetal male cortices were subjected to quantitative proteomic analysis using isobaric tags for relative and absolute quantitation (iTRAQ). Male fetuses were used due to their known preferential sensitivity to cannabis [81,90]. Proteomics analysis yielded 35 DEPs that are involved in structural activity/cytoskeleton, protein biogenesis, RNA metabolism, cell adhesion, metabolism and chromatin organization pathways. Amongst these DEPs, superior cervical ganglion 10 (SCG10) protein was noticeably decreased, and these findings were also corroborated in human fetal samples. SCG10 is a microtubule-binding protein with a key role in modulating cytoskeletal dynamic instability required for axonal guidance and arborization. The activity of SCG10 is regulated by c-Jun N-terminal kinase (JNK) which is a downstream target of CB1R. These reports suggest that THC impaired axonal morphology and neurite outgrowth is potentially mediated via CB1R activation and subsequent effects on JNK dependent SCG10 degradation [79,89,90].

In addition to prenatal findings, cumulative evidence suggests that adolescents are susceptible to both short- and long-term side effects of cannabis [91,92,93,94]. Considering the importance of the ECS in adolescent brain development and synaptic plasticity, alterations in neuronal cell proliferation, migration, and differentiation during these stages may lead to long-term adverse effects [95,96,97,98]. In this regard, Quinn and colleagues (2008) employed MALDI-TOF mass spectrometry to compare the adverse effects of cannabis on adolescent and adult rats. Their results revealed that adolescent rats (>28 post-natal days) are preferentially affected by THC long-term adverse effects when compared to adult animals (>60 post-natal days), even after being subjected to 10–15 days washout cycle. Additionally, significant impairment in object recognition and memory was observed in adolescent rats when compared to adult animals. Hippocampal proteome analysis after 17 days (when no residual THC was detected in blood) identified 27 DEPs in THC-treated adolescent rats when compared to adolescent controls. These DEPs were mainly mitochondrial and cytoskeletal proteins such as heat shock cognate 71 kDa protein (HSP7C), mitochondrial stress-70 protein (GRP75), and transgelin-3 (TAGL3). In adult rats, only 10 DEPs were observed in the treated group when compared to controls, and these were primarily implicated in regulating mitochondrial/metabolic activity, such as mitochondrial aconitate hydratase (ACON) and glyceraldehyde-3-phosphate dehydrogenase (G3P) pathways [76]. This study also demonstrated that adolescents are more susceptible to long-term adverse effects of cannabis than adults, highlighting that mitochondria and cytoskeleton may be primary targets of biological effects of cannabis-related components [76]. In order to explore the long-term adverse effects of marijuana use during adolescence, Filipeanu and colleagues (2011) performed a proteomic analysis on cerebellar extracts obtained from adult female rats (261 PND) that were exposed to THC for 40 days during adolescence (35 to 75 post-natal days). Only six DEPs were identified, four of which were decreased, including two mitochondrial proteins, pyruvate carboxylase (PYC) and NADH dehydrogenase (NDUAA). The other identified proteins were the cytosolic protein nucleoside diphosphate kinase B (NDKB), which is predominantly involved in cell adhesion and migration processes, as well as translationally controlled tumor protein (TCTP), which is a calcium binding protein involved in microtubular stabilization. The study also identified two proteins which were increased including Parkinson’s disease protein 7 (PARK7), which plays an important role in cell protection against oxidative stress and cell death, as well as the 90 kDa activator of heat shock protein ATPase homolog 1 (AHSA1), a co-chaperone for HSP90 that increases its chaperone activity [79]. These findings highlighted impaired mitochondrial biogenesis and cytoskeleton in the cerebellum as long-term effects of cannabis abuse during adolescence. In another study, Spencer and colleagues (2013) reported changes in oxidative stress and calcium-binding proteins including glutathione d-transferase mu 2 (GSTM2), heat shock 70 kDa protein 4 (HSPA4), calretinin (CALB2) and ADP-ribosylation factor-like protein 1 (ARL1) in the hippocampus of male mice that were treated with THC during adolescence (31 to 52 days post-natal) [80]. With respect to these findings, Rubino and colleagues (2009) showed that exposure to THC during adolescence (35 to 45 post-natal days) caused long-term adverse effects on memory in female rats. To examine the association of memory impairment with neuroplasticity, levels of neural plasticity markers such as synaptophysin (SYPH) and postsynaptic density-95 (PSD95) were measured. While no changes were observed in the hippocampal tissue, significant down-regulation of both proteins was observed in the prefrontal cortex, suggesting region-specific variation in the brain. Proteome analysis of synaptosomes in this region from the THC-treated group yielded 11 DEPs, which were mainly mitochondrial proteins including Cytochrome b-c1 complex subunit 1 and subunit 2 (QCR1 and QCR2) and ATP synthase subunits, as well as various proteins involved in metabolic pathways [78]. Mitochondrial proteome changes were also reported in another study that investigated the proteomic changes in the cerebellum of adult mice (2 months) upon chronic exposure (4.5 days) to THC. The results of this study by Colombo and colleagues (2009) showed alteration of seven DEPs in the cerebellum cytosolic fraction and 24 DEPs in the cerebellum membrane fraction. Amongst these, three main protein classes were identified including guanine nucleotide-binding proteins (G-proteins), calcium-binding proteins and membrane transcytosis-related proteins. These proteins included guanine nucleotide-binding protein beta subunit 5 (GNB5), calretinin (CALB2), hippocalcin-like protein 1 (HPCL1) and vesicle Protein Sorting 29 (VPS29), all of which were significantly up-regulated. The down-regulation of various RNA/DNA binding and mitochondrial proteins was also evident, such as in case of Non-POU domain-containing octamer-binding protein (NONO), heterogeneous nuclear ribonucleoprotein K (HNRPK) and NADH-ubiquinone oxidoreductase 75 kDa subunit (NDUS1) [77].

CB1Rs are mainly expressed in presynaptic terminals and play a role in neurotransmitter release, therefore exploring protein changes in synaptosomes can provide valuable insights into the effects of THC [99,100]. Salgado-Mendialdúa and colleagues (2018) performed Tandem Mass Tag (TMT) labeling to explore the proteome changes in mice hippocampal synaptosomal fractions in mice administered THC intraperitoneally. Three hours post-administration, their analysis showed the alteration of 122 proteins, 112 of which were synaptic proteins and mainly related to cytoskeletal reorganization and metabolic pathways, particularly mitochondrial dysfunction. Moreover, altered proteasome system activity such as the significant down-regulation of 20S proteasome chymotrypsin-like protease was observed, which may underlie impairment of synaptic plasticity in the THC treated groups [82,101,102].

Recent studies have also highlighted the up-regulation of mitochondrial proteins upon THC application. A recent iTRAQ proteomics study by Beiersdorf and colleagues (2020) established that the daily administration of THC during the early postnatal period adversely affected neuronal bioenergetics and neuronal survival [83]. Proteome profiling of the hippocampus of pre-adolescent mice (pp. 5–35; mice preadolescent period) demonstrated a total of 33 DEPs, mainly involved in protein synthesis, cytoskeletal modifications and cell adhesion required for neurite outgrowth. THC administration was completed at P35 and was followed by two washout periods at P48 (14 days) and P120 (85 days) to delineate long-term changes in the hippocampus proteome. P48 demonstrated 31 DEPs including the up-regulation of respiratory-chain (Atp5h, Atp6v1e1), antioxidant (Prdx1/2, Sod2), and ATP synthesis–related proteins (Nme2). It was suggested that increased protein expression may be a compensatory response to THC exposure, which adversely affected neuronal bioenergetics. Additionally, P120 showed 186 DEPs, 49 of which were up-regulated mitochondrial proteins, indicating long-lasting effects of THC on neuronal bioenergetics [83,103,104]. Collectively, the results of the proteomic studies discussed above show dysfunctional neuronal mitochondrial biogenesis, as well as impaired neuronal cytoskeleton growth and development, as the main consequences of THC toxicity.

## 4. Proteomic Studies on Cannabidiol

CBD was reported to have milder psychoactive properties and fewer side effects than THC and a growing number of studies have reported its beneficial effects in comparison to the adverse effects associated with THC [66,105]. Therefore, CBD appears to be a desirable agent for medical purposes, yet only a limited number of proteomic studies have been performed thus far. The majority have investigated its beneficial effects on the skin, as noted by Casares and colleagues (2020) [106]. Their study determined the proteome changes in primary human keratinocytes following 24h exposure to CBD. Their analyses yielded 724 DEPs, of which 520 and 204 proteins were down- and up-regulated, respectively. Transcriptomic analysis to study gene expression revealed that 147 out of 724 DEPs showed the same pattern of alteration at mRNA levels. Amongst these, the proteins up-regulated have been implicated in the proliferation and differentiation of keratinocytes and skin development, as well as proteins with antioxidant activity such as Nuclear factor erythroid 2-related factor 2 (NFE2L2) and Heme oxygenase 1 (HMOX1). Meanwhile, down-regulated proteins were more associated with regulating the organization of the extracellular matrix, DNA metabolic processes and cell cycle [106]. This study indicated that CBD treatment can potentially enhance antioxidant activity and increase keratinocyte proliferation, which suggests that CBD could be used as a potential treatment for several skin conditions.

Since human skin is constantly exposed to ultraviolet radiation (UVR), the beneficial effects of CBD were also previously investigated on nude rat skin exposed to ultraviolet A/B (UVA/UVB) radiation (365/312 nm). Human Phototherapy with UV has been an effective treatment option for several chronic skin disorders such as psoriasis [107], however, it has adverse effects on skin cell function and survival, leading to several side effects ranging from erythema (sunburn) to skin cancer [108,109]. Keratinocyte hyperproliferation (accounting for 90–95% of the epidermal cell population), inflammation and apoptosis have been repeatedly reported upon UV exposure [107,110]. Atalay and colleagues (2021) showed that UVA and UVB exposure caused the alteration of 54 and 47 proteins, mostly involved in oxidative stress response, inflammation and apoptosis. These include NFE2L2, interleukin 18 receptor (IL18R) and transforming growth factor-beta (TGF-β), which were strongly attenuated in the CBD treated group. These findings indicate that the topical administration of CBD can maintain keratinocyte proteostasis even upon exposure to UV rays [111].

To study these observed beneficial effects of CBD on human skin, researchers performed two further studies on human keratinocytes cultured in two- and three-dimensional (3D) systems [112,113]. Three-dimensional cell culture efficiently resembles the in vivo environment and provides valuable insight into the effects of CBD on human skin [60,114]. Three-dimensional cell culture showed that exposing cells to UVA and UVB altered the expression of 456 and 217 proteins, respectively. Most of these proteins were involved in the inflammatory response, including regulating tumor necrosis factor (TNFα) and nuclear factor NF-kappa-B p105 subunit (NFκB). CBD significantly decreased the expression of these proteins, such as heat shock protein HSP 90-beta (HS90B) and hsp90 co-chaperone Cdc37 (CDC37), in the UVA exposed group, but not in the UVB group. Moreover, CBD efficiently prevented UVA/B induced alterations in the lipid peroxidation level and the production of 4-hydroxynonenal (4-HNE) which adversely affects antioxidant enzymes [115,116]. However, no significant alterations in the expression of 40S or 60S ribosomal proteins and proteasome proteins were observed, which were significantly altered following exposure to UVA/B [113]. These results highlighted the anti-inflammatory and antioxidant effects of CBD on epidermal cells and indicate CBD as a potential therapeutic option to minimize the harmful effects of ultraviolet radiation.

## 5. Proteomic Studies on Other Cannabis-Related or Derived Compounds

As previously mentioned, approximately 100 cannabinoids are produced by cannabis, and the majority of research has focused on THC and CBD. One compound of interest that is derived from cannabis is Cannabigerol (CBG); such as CBD it has a slight affinity for CB1R and CB2R, but showed higher affinity for CB2R [117,118,119,120], although its detailed mechanisms of pharmacological actions are not yet fully understood. A recent proteomic study compared the therapeutic effects of CBD and CBG in a rat astrocytic culture and in isolated rat cortices, mimicking pathophysiological events of migraine, hypoxia/ischemia and epilepsy [121]. The study demonstrated that both CBD and CBG effectively restored the expression of histone H2B, which is involved in transcription regulation and replication, and repair of DNA. However, only CBD was able to restore the expression of three synaptic proteins involved in synaptic plasticity, including synaptotagmin-1 (Syt 1), syntaxin 1b (Stx 1b) and calcium/calmodulin-dependent protein kinase type II subunit alpha (CAMK2A) [121]. Although the results of this study indicated that CBG at lower concentration compared to CBD showed the same antioxidant effects, CBD showed beneficial effects on neurotransmitter exocytosis, which was not observed in CBG, suggesting that CBD may be a more desirable therapeutic option. Currently, CBD is an FDA-approved treatment option against two rare and severe types of epilepsy including Lennox–Gastaut syndrome (LGS) and Dravet syndrome, in patients two years of age and older [122,123].

Besides natural cannabis-derived compounds, synthetic cannabinoids (SCs) such as cyclohexylphenols have been developed and marketed worldwide. One particular cyclohexylphenol, known as (CP-47,497-C8) is an SC that binds to both CB1 and CB2 cannabinoid receptors with a higher preference for CB1R [124,125]. It was widely used as a legal substitute for cannabis, however, there is limited research investigating its cytotoxic effects [126,127]. A proteomic study by Bileck and colleagues (2016) examined the effects of this synthetic cannabinoid on the nuclear proteome of lymphocytes from four healthy men aged between 25 and 32 years. A dysregulation of 249 proteins in the CP-47,497-C8 treated group was observed compared to the control. The up-regulated proteins were mainly involved in metabolic processes, signal transduction in DNA damage, and antigen processing and presentation, while the down-regulated proteins were primarily related to macromolecule biosynthesis, RNA splicing, and transcription cofactor activity. Ingestion of this SC was also associated with increased DNA damage, due to the inhibitory effects on proteins involved in DNA repairs such as excision repair 5 exonuclease (ERCC5) and double-strand break repair protein (MRE11A). Tissue analysis also revealed enhanced inflammation levels, as demonstrated via the up-regulation of pro-inflammatory cytokines, such as interleukin-8 (IL-8), tryptophan tRNA ligase (SYW) and signal transducer and activator of transcription 5A (STAT5A) [126]. Taken together, this study indicates increased DNA damage and inflammation as the major adverse effects associated with CP-47,497-C8 use. Adverse effects of synthetic cannabinoids can also be found in a study by Scherma and colleagues (2020), that explored the synaptosomal and cytosolic proteome changes in the prefrontal cortex of adolescent male mice that were exposed to the synthetic cannabinoid WIN 55,212-2 (WIN) for 11 consecutive days (42 to 53 post-natal days). Their proteomics analysis yielded about 755 and 274 DEPs in synaptosomal and cytosolic fractions respectively. Further analysis using DEPs from both fractions showed that up-regulated proteins were mainly involved in NLS-bearing protein import into nucleus, ER to Golgi vesicle-mediated transport, tRNA metabolic process and import into nucleus, while down-regulated proteins had a role predominantly in the tricarboxylic acid cycle, long-term synaptic potentiation, protein localization to synapse and regulation of synaptic plasticity. Interestingly, pre-exposure to WIN during adolescence but not adulthood was shown to induce molecular and epigenetic changes following exposure to cocaine and resultant enhanced response to cocaine’s stimulatory effects [84]. Association of psychoactive cannabinoid use during adolescence and increased behavioral effects of cocaine have been repeatedly reported [84,128,129,130]. Future studies should investigate the neurobiological consequences of cannabis use during critical developmental stages such as in adolescence to guide legislation and public health policy.

## 6. Conclusions

Cannabis use has seen a consistent increase worldwide, which emanates from its observed therapeutic properties and increased social acceptance, despite the fact that molecular effects of its long-term usage on CNS remain shrouded in mystery. It is anticipated that recent legislative modifications in cannabis use policy will facilitate research that can provide in-depth empirical evidence on both the therapeutic and toxic effects of cannabis. Proteomic studies, though limited in number, have proven to be an efficient tool to delineate the effects of cannabis-derived or related compounds on cells and tissues. Future proteomic research should consider the potential effects of confounding factors such as genetic background, age, gender, and the difference between acute and chronic use, to best explore the short- and long-term effects of cannabis on human health. Additionally, considering the impact of post-translational modifications such as phosphorylation and glycosylation on protein function, exploring changes in post-translational modifications along with proteome alteration would yield valuable insights into the mechanisms underlying the therapeutic and toxic effects of cannabis.

## Figures and Tables

**Table 1:** Summary of proteomic studies on effects of cannabinoid exposure on the brain.

Study	Sample Type	Cannabis Exposure	Method	Number of DEPs	Significant Enriched Pathways	References
Bindukumar et al., 2008	Normal human astrocytes	THC (1 × 10^−7^ M) for 48 h	2D-DIGE–LC-MS/MS	24 proteins	Glycolysis, binding and folding, kinase, and molecular chaperone	[75]
Quinn et al., 2008	Hippocampus of adolescent (PND28) and adult (PND60) male rats	THC injection (1 mg/kg) for 2 consecutive days following by 8 doses injection (5 mg/kg) on alternate days	2D-DIGE–MALDI-TOF MS	27 proteins in adolescent and 10 proteins in adult	Mitochondrial, cytoskeletal and metabolic proteins	[76]
Colombo et al., 2009	Cerebellum of adult mice (2-months-old)	THC twice a day injection (10 mg/kg) for 4.5 days	2D-DIGE–ESI-MS/MS	31 proteins	Guanine nucleotide-binding proteins (G proteins), calcium-binding, exo–endocytosis, RNA/DNA binding and mitochondrial proteins	[77]
Rubino et al., 2009	Prefrontal cortex of adult (PND75) female rats	THC injection twice a day in adolescence (2.5 mg/kg PND 35–37; 5 mg/kg PND 38–41; 10 mg/kg PND 42–45)	2D-DIGE–MALDI-TOF MS	11 proteins	Mitochondrial proteins, glycolysis and molecular chaperone	[78]
Filipeanu et al., 2011	Cerebellum of adult female rats	THC daily injection (5.6 mg/kg) from PD 35 to PD 75	2D-DIGE–LC-MS/MS	6 proteins	Cellular energy metabolism (mitochondrial), cell adhesion, migration and cytoprotection	[79]
Spencer et al., 2013	Hippocampus of male mice (52PND)	THC daily injection (10 mL/kg) for 21 days	2D-DIGE–MALDI-TOF MS	4 proteins	Oxidative stress, calcium signaling, and innate immune response	[80]
Tortoriello et al., 2014	Hippocampus of male fetal mice (E18.5)	Pregnant mice were injected THC (3 mg/kg) from E5.5 to E17.5 daily	iTRAQ–nLC-ESI/MS/MS	35 proteins	Structural activity/cytoskeleton, protein biogenesis, RNA metabolism, cell adhesion, metabolism and chromatin organization, signaling	[81]
Salgado-Mendialdúa et al., 2018	Hippocampus of 3-months-old male C57BL/6J mice	Single THC (10 mg/kg) injection	TMT–nLC-MS/MS	122 proteins	Metabolic pathways (mitochondrial), cytoskeletal reorganization pathways, proteasome system	[82]
Beiersdorf et al., 2020	Hippocampus of preadolescent male mice	THC daily injection (1 mg/kg or 5 mg/kg) from PD5 to PD35	iTRAQ–nLC-ESI/MS/MS	31 proteins	Mitochondrial function, cytoskeletal rearrangement, RNA turnover, chromatin modifications	[83]
Scherma et al., 2020	prefrontal cortex of adolescent male mice	WIN 55,212-2 daily injection (2–8 mg/kg) from PD 42 to PD 53	TMT-nLC–MS/MS	1029 proteins in two fractions (755 in synaptosomal and 274 in cytosolic fractions)	NLS-bearing protein import into nucleus, ER to Golgi vesicle-mediated transport, tRNA metabolic process, import into nucleus, tricarboxylic acid cycle, long-term synaptic potentiation, protein localization to synapse and regulation of synaptic plasticity	[84]
Delgado-Sequera et al., 2020	ON cells of either sex chronic cannabis users	Plasma concentration: THC-COOH: 29.76 ± 6.15 ng/mL	LC-MS/MS	65 proteins	Cytoskeleton (particularly microtubule dynamics and its influence on cell morphology), cell proliferation and growth (e.g., outgrowth of neuritis) and apoptosis	[69]
Barrera-Conde et al., 2021	ON cells of either sex chronic cannabis users	Plasma concentration: THC-COOH: 34.92 ± 17.55 ng/mL	SWATH-MS	102 proteins	Immune system, RNA metabolism, cellular responses to externalstimuli, protein localization	[70]
Xiao et al., 2021	Hippocampus of 8-months-old male mouse models of AD	THC 400 (mg/kg) for 5 months (i.g.)	nUHPLC/NSI–MS/MS	157 proteins	IFNγ production, T cell activation, lymphocyte activation, response to bacterium, protein oligomerization, protein binding, assembly and organization of membrane raft.	[85]

**Abbreviations:** PND/PD, post-natal days; E, embryonic days; DEP, differentially expressed proteins; ON, olfactory neuroepithelium; (i.g.), intragastrically.

## Data Availability

Not applicable.
